# Acute abdominal pain as the first symptom of *Chlamydia psittaci* pneumonia complicated by acute pancreatitis: a case report

**DOI:** 10.3389/fmed.2023.1253859

**Published:** 2023-10-11

**Authors:** Changquan Fang, Yanjun Xie, Hui Mai, Limin Xu

**Affiliations:** ^1^Department of Pulmonary and Critical Care Medicine, Huizhou Central People’s Hospital, Huizhou, Guangdong, China; ^2^Department of Geriatrics, Huizhou First People’s Hospital, Huizhou, Guangdong, China

**Keywords:** psittacosis, *Chlamydia psittaci* pneumonia, acute pancreatitis, abdominal pain, metagenomic next-generation sequencing

## Abstract

**Background:**

*Chlamydia psittaci* infections primarily cause damage to the lungs but may also affect the cardiovascular system, gastrointestinal tract, liver, kidney, and brain, resulting in a variety of extrapulmonary complications. However, reports regarding *C. psittaci* infection-associated pancreatitis are rare. In this report, a patient with *C. psittaci* pneumonia complicated by acute pancreatitis is presented.

**Case description:**

The patient presented with acute upper abdominal pain and developed severe pyrexia and dyspnoea one day later. A chest computed tomography image revealed patchy consolidation in the left lung. The disease progressed rapidly, and the patient exhibited liver and kidney damage and type 1 respiratory failure within a short period of time. Metagenomic next-generation sequencing of alveolar lavage fluid revealed the presence of *C. psittaci*. The patient was administered doxycycline and moxifloxacin, after which the patient’s abdominal pain and lung infection significantly resolved.

**Conclusion:**

This case report demonstrates that extrapulmonary *C. psittaci* infections due to secondary acute pancreatitis can manifest as abdominal pain, although the exact mechanisms of *C. psittaci* caused by acute pancreatitis remain unclear. Timely diagnoses and treatments of such infections are necessary to achieve favorable clinical outcomes.

## Introduction

*Chlamydia psittaci* is a rare respiratory pathogen, responsible for approximately 1% of cases of community-acquired pneumonia (1). *C. psittaci* often induces various extrapulmonary complications, such as acute liver injury, acute renal failure, myocarditis, and meningitis (2). However, the occurrence of secondary acute pancreatitis in patients with *C. psittaci* pneumonia has rarely been reported (3). Common symptoms of psittacosis include severe pyrexia, chills, headache, dyspnea, and cough. Prodromal symptoms of nausea, vomiting, and abdominal pain occur in certain patients (4). The lack of specificity in the clinical manifestations of psittacosis limits the use of traditional pathogen detection methods, including pathogen cultures, serological tests, and the polymerase chain reaction, resulting in inadequate or delayed diagnoses (2). Metagenomic next-generation sequencing (mNGS) is superior to traditional detection methods in terms of its ability to identify suspected pathogenic microbes within samples in a rapid, efficient, and accurate manner. It is an effective detection method for the diagnosis of *C. psittaci* infections (5). Few cases of psittacosis complicated by acute pancreatitis have been reported. This is a report of a patient with *C. psittaci* pneumonia complicated by acute pancreatitis who presented with acute abdominal pain. The aim of this report is to enhance clinicians’ knowledge regarding this disease.

## Case description

**Figure 1:**
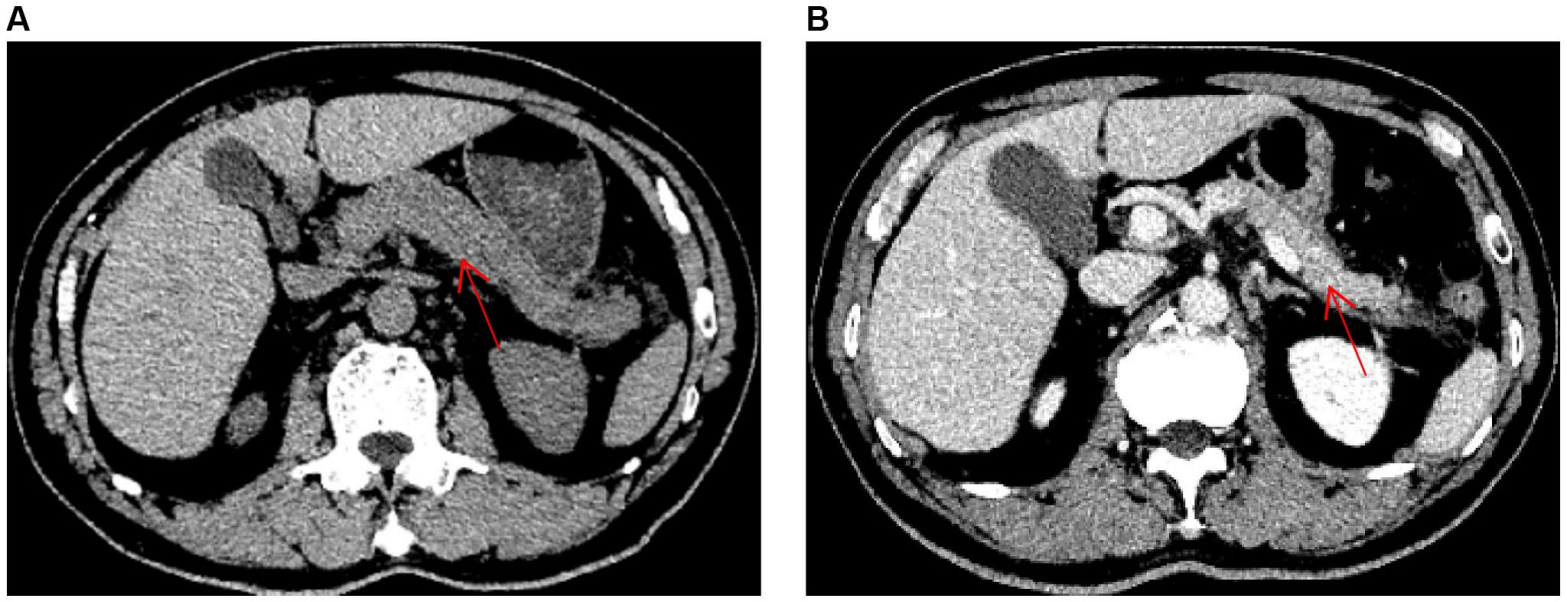
Abdominal computed tomography images (red arrow indicates pancreas). **(A)** An abdominal computed tomography image obtained on hospital day 1, prior to treatment, is shown. **(B)** An abdominal computed tomography image obtained on hospital day 10, after treatment, is shown.

A 69-year-old man presented with upper abdominal pain that radiated to his back and was accompanied by nausea and vomiting. The patient had no history of underlying diseases and did not smoke or drink alcohol. He reported using no medications within one month of the onset of pain. Upon presentation, the patient’s blood amylase and lipase concentrations were 916 U/L and 53 U/L, respectively. The patient’s myocardial enzyme concentrations and electrocardiographic results were normal. Abdominal computed tomography (CT) revealed mild swelling and thickening of the pancreas, blurring of the lipid spaces around the pancreas, and no gallbladder or extrahepatic bile duct expansion (Figure 1A). A diagnosis of acute pancreatitis was considered. The patient fasted and was administered symptomatic and supportive therapies, including analgesics, hydration, and antibiotic treatment with levofloxacin (500 mg qd) intravenously. However, his abdominal pain did not subside, and the patient was admitted to the gastroenterology department for further diagnosis and treatment.

Upon admission, the patient’s temperature was 36.9°C, heart rate was 82 beats/min, respiratory rate was 26 breaths/min, blood pressure was 113/70 mmHg, and oxygen saturation was 94%. The patient was fully conscious and had no enlarged superficial lymph nodes. Upon auscultation, he had mild shortness of breath, bilateral coarse breath sounds, and no dry or wet rales, and his heart rhythm was normal, with no pathological murmurs. The patient’s abdomen was soft, with tenderness below the xiphoid process and in the left upper quadrant, with no rebound tenderness. He did not exhibit oedema in his lower extremities.

**Figure 2:**
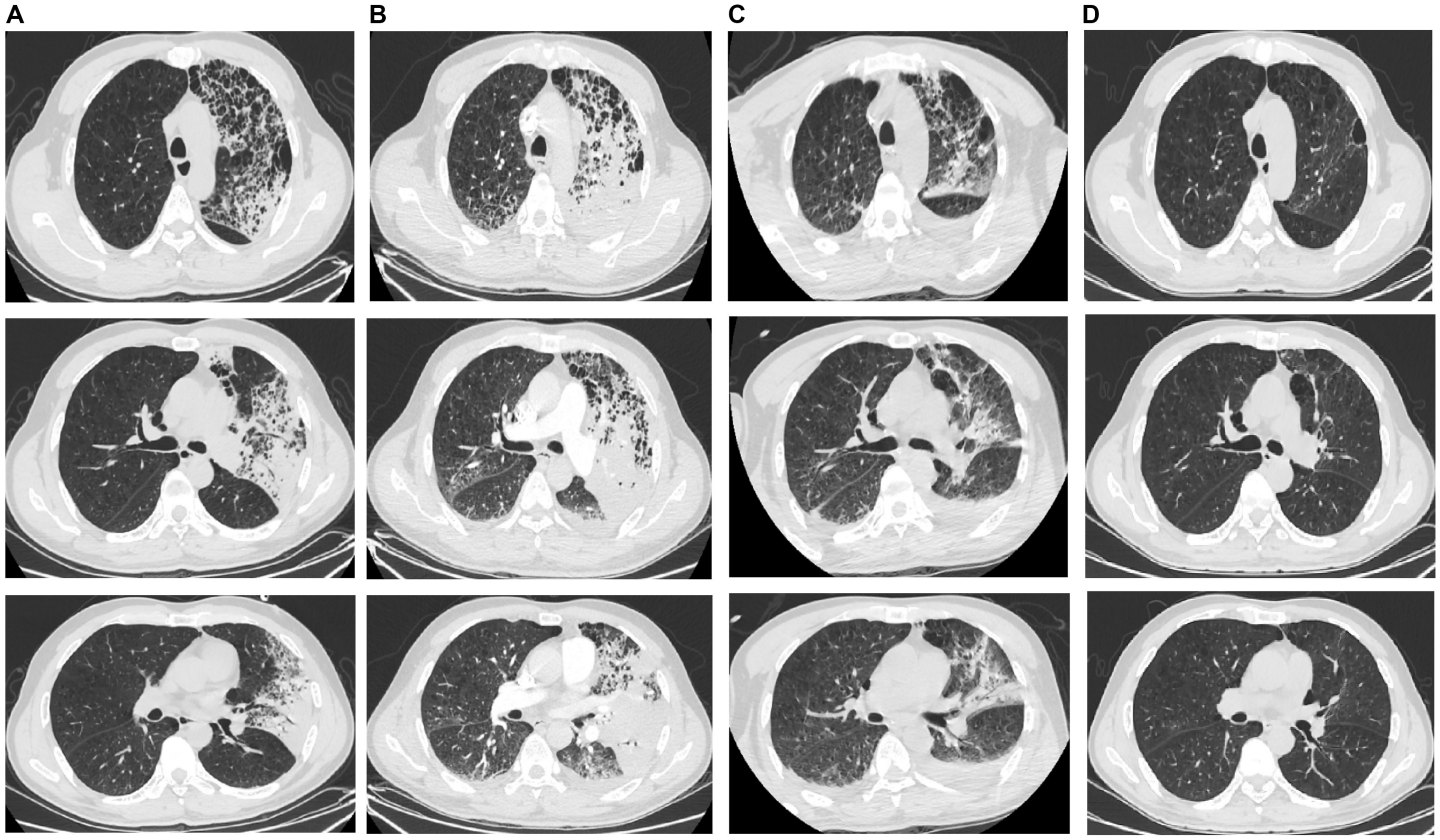
Chest computed tomography images **(A)** A chest computed tomography image obtained on hospital day 2 shows left upper-lobe patchy consolidation shadows. **(B)** A chest computed tomography image obtained on hospital day 3 shows increased consolidation shadows in the left lung and a small bilateral pleural effusion. **(C)** A chest computed tomography image obtained on hospital day 15 shows marked resorption of the left lung lesion and a small increase in the bilateral pleural effusion. **(D)** A chest computed tomography image obtained on hospital day 27 shows that the lung lesions are resolved.

Once admitted, the patient was administered octreotide to inhibit pancreatic enzyme secretion and an esomeprazole injection to suppress gastric acid secretion, upon which his abdominal pain was slightly alleviated. However, on hospital day 2, he developed pyrexia (39.5°C) with chills, fatigue, headache, and muscle aches. The patient experienced dyspnea upon physical exertion, and a chest CT revealed patchy consolidation in the superior lobe of the left lung (Figure 2A). A blood gas analysis revealed a fraction of inspired oxygen of 33%, pH of 7.49, partial pressure of oxygen of 65.0 mmHg, partial pressure of carbon dioxide of 26.4 mmHg, and bicarbonate concentration of 18.7 mmol/L. The patient’s total blood cholesterol concentration was 4.21 mmol/L, triglyceride concentration was 1.72 mmol/L, and serum calcium concentration was 2.15 mmol/L. Other laboratory test results are summarized in Table 1. The patient was diagnosed with severe community-acquired pneumonia complicated by acute pancreatitis.

**Table 1:** Results of laboratory tests of the patient at different times.

Laboratory test	Normal range	First day of hospitalization	After 3 days of targeted antibiotic therapy	One day before discharge
*Blood routine*				
WBC (×10^9^/L)	4–10	9.0	7.7	6.2
Neutrophil (%)	40–75	94.1	69.6	62.5
LYM (×10^9^/L)	1.1–3.2	0.5	0.79	1.42
*Inflammatory index*				
C-reactive protein (mg/L)	0–5	313.2	120.8	4.92
Procalcitonin (ng/mL)	0–0.05	15.6	2.5	0.03
Interleukin-6 (pg/mL)	0–7	380.2	164.1	5.7
*Biochemical indexes*				
Amylase (U/L)	35–135	916	361	73
Lipase (U/L)	13–60	53	261	40
ALT (U/L)	9–50	126	65	13
AST (U/L)	15–40	222	85	16
CK (U/L)	50–310	661	338	90
LDH (U/L)	109–245	573	350	134
D-dimer (mg/L)	0–500	17,440	5,310	479
Scr (μmol/L)	62–106	148	106.5	79.0
BNP (pg/mL)	0–100	136.3	91.2	63.5
High-sensitivity troponin T (ng/mL)	14–100	18.7	10.1	8.4

ALT, alanine aminotransferase; AST, aspartate aminotransferase; BNP, brain natriuretic peptide; CK, creatine kinase; LDH, lactate dehydrogenase; LYM, lymphocyte count; Scr, serum creatinine; WBC, white blood cell.

On hospital day 2, the patient was transferred to the Department of Respiratory and Critical Care Medicine. He was administered meropenem (1.0 q8h) intravenously and moxifloxacin (400 mg qd) intravenously as anti-infective therapy, umifenovir (200 mg tid) as antiviral therapy, and nasal high-flow oxygen therapy for acute pancreatitis. However, the patient continued to have severe pyrexia and his abdominal pain did not considerably resolve. CT pulmonary angiography revealed increased consolidation in the left lung and a small degree of bilateral pleural effusion (Figure 2B). The patient’s blood amylase concentration was 1,150 U/L, blood lipase concentration was 324 U/L, urinary amylase concentration was 1,742 U/L, white blood cell count was 6.7 × 10^9^/L, lymphocyte count was 0.28 × 10^9^/L, neutrophil percentage was 97.6%, C-reactive protein concentration was 280 mg/L, and procalcitonin concentration was 11.3 ng/mL. As the patient did not respond to conventional antibiotic treatment, the possibility of infection with a special pathogen was suspected. On hospital day 4, fibreoptic bronchoscopic examination revealed no obvious abnormalities. Alveolar lavage fluid (10 mL) was obtained for microbe identification via mNGS. The patient’s serum antinuclear antibody, antineutrophil antibody, and immunoglobulin (Ig) concentrations were within the normal ranges. The patient was negative for severe acute respiratory syndrome coronavirus 2 nucleic acid, influenza virus nucleic acid, serum 1,3-β-D-glucan, galactomannan, and IgM antibodies against respiratory pathogens (*Legionella pneumophila*, *Mycoplasma pneumoniae*, *Rickettsia* spp., *Chlamydia pneumoniae*, adenoviruses, respiratory syncytial virus, influenza A virus, influenza B virus, and parainfluenza viruses). His blood and sputum cultures were also negative.

On hospital day 6, the mNGS results indicated the presence of *C. psittaci* (number of sequences: 512, relative abundance: 90.3%, coverage: 91%) and *Candida albicans* (number of sequences: 698, relative abundance: 57.8%, coverage: 98%). At that time, the patient divulged that he had found and buried several dead chickens in his vegetable field 10 days prior to the onset of symptoms. Therefore, he was diagnosed with severe *C. psittaci* pneumonia, and his treatment regimen was changed to a combination of moxifloxacin (400 mg qd) intravenously and doxycycline (100 mg q12h) on hospital day 6. On hospital day 9, the patient’s body temperature returned to normal, and his abdominal pain and dyspnea were substantially resolved. His oxygenation index and markers of inflammation and organ functions were also improved (Table 1). Abdominal CT revealed a reduction in the pancreatic swelling and in the patchy, blurred, low-density shadows around the pancreas (Figure 1B). On hospital day 11, the patient’s abdominal pain was relieved, and his blood amylase concentration was normal. The patient was started on a bland liquid food diet, which was tolerated. On hospital day 14, chest CT revealed marked resorption of the consolidation in the left lung and a slight bilateral increase in pleural effusion (Figure 2C). The nasal high-flow oxygen therapy was replaced with oxygen inhalation via a nasal cannula. On hospital day 26, chest CT revealed no exudative lesion of the left lung and no pleural effusion (Figure 2D). Markers of inflammation and various organ functions had generally returned to normal (Table 1). The patient was discharged on hospital day 27. Doxycycline (100 mg q12h) was continued for one week. At a one-month follow-up visit, the patient was in a generally good condition with an occasional cough without dyspnea, abdominal pain, or diarrhea.

## Discussion and conclusions

*C. psittaci* is a gram-negative, obligate intracellular parasitic pathogenic microbe that mainly relies on the mononuclear macrophage system for its growth and metabolism. Its intracellular parasitic ability enables it to evade immune attacks by the host. This pathogenic bacterium releases endotoxins, causing the body to produce autoimmune and allergic reactions, and it directly damages host cells. During the anti-*Chlamydia* immune response, T cells and infection-related cells further aggravate the immune damage, ultimately resulting in multiple organ dysfunction (6, 7). The clinical symptoms of patients with psittacosis lack specificity and can range in severity, from mild influenza-like symptoms to systemic infection mainly presenting as atypical pneumonia. Common extrapulmonary complications include hepatitis, meningitis, acute renal failure, and myocarditis (2, 8). In this case, the patient’s first symptom was acute abdominal pain due to *C. psittaci*-related acute pancreatitis, which is rare.

Acute pancreatitis is a common critical disease of the digestive system. It arises as a consequence of the activation of pancreatic enzymes and is characterized by local inflammation of the pancreas. Common etiologies of pancreatitis include gallstones, excessive alcohol use, and hypertriglyceridemia. Pancreatitis may also be caused by pancreatic tumors, hypercalcemia, medication, infection, and systemic inflammatory responses, although these etiologies are uncommon in clinical practice (9, 10). The patient in this report had no history of alcohol use, medication use, or biliary diseases. No biliary tract stones, bile duct dilatation, or pancreatic space occupation was noted on abdominal CT, and the patient’s concentrations of triglycerides, blood calcium, and autoimmune disease indicators were within the normal ranges. That, combined with the fact that his pancreatitis was significantly alleviated after targeted anti-psittacosis treatment, led to a diagnosis of acute pancreatitis secondary to *C. psittaci* infection.

Acute pancreatitis can be caused by a variety of pathogens, including severe acute respiratory syndrome coronavirus 2, *M. pneumoniae*, and *Legionella* spp. (11–13). However, reports of secondary acute pancreatitis in patients with a *C. psittaci* infection are rare (3), which may be owing to the low incidence of psittacosis, difficulty of its diagnosis, or lack of clinician awareness. Byrom et al. (14) first reported two cases of *C. psittaci* pneumonia with concomitant acute pancreatitis in 1979. Both patients presented with digestive symptoms and pyrexia and ultimately died of the disease. The diagnosis was confirmed via serological examination in one case and autopsy in the other. Additional cases have been reported since. The patient in this report had concomitant pneumonia and acute pancreatitis recalcitrant to conventional methods. The causative pathogen was subsequently detected using mNGS, which directed treatment and led to a favorable clinical outcome. A diagnosis of psittacosis relies primarily on laboratory tests, including pathogen isolation and culturing, serological tests, polymerase chain reaction tests, and mNGS. Pathogen isolation and culturing require high laboratory qualifications and carry the risk of contamination; serological tests are time-consuming and unsuitable for early diagnosis; and, although polymerase chain reaction tests are sensitive and specific, they are susceptible to missing a diagnosis because a limited number of pathogens can be screened per test (15, 16). Furthermore, most hospitals in China are not equipped to routinely perform such tests. mNGS is not subject to culturing and requires no prior assumptions in terms of the causative bacteria. Moreover, it can be used for direct high-throughput sequencing of nucleic acids in clinical samples toward identification of unknown pathogens, pathogens causing mixed infections, and pathogens that are difficult to culture (17, 18). As *C. psittaci* is an absolute pathogen, it does not typically exist in human samples, and even a small number of detected sequences has diagnostic value (19). The mechanisms by which psittacosis causes pancreatic damage remain unclear. Although *C. psittaci* infection causes hypoxia and systemic inflammatory responses, it may also inflict direct damage on the pancreas, as *C. psittaci* infection has been observed in the pancreas during animal and human autopsies (14, 20).

The main focus of the treatment of *C. psittaci* pneumonia complicated by acute pancreatitis is the early recognition of pancreatitis and its complications and the timely administration of treatments, such as fasting, inhibition of pancreatic juice secretion, and hydration (10). However, the early identification of the causative pathogen and provision of targeted treatment before the occurrence of multiple organ failure are of greater importance (14). Tetracyclines are the most common treatment for *C. psittaci* pneumonia, followed by macrolides and respiratory quinolones. Medication should be administered for at least three weeks to prevent disease recurrence (2). The initial administration of moxifloxacin in the current patient was ineffective, which might have been due to the relatively low intracellular antibacterial activity of quinolones (21). Previous reports indicate that tetracyclines and macrolides may cause acute pancreatitis (10, 22, 23). Therefore, the blood amylase concentration should be monitored while they are administered. Our patient received doxycycline after the onset of acute pancreatitis and did not suffer aggravation of his pancreatitis during the course of treatment, which excluded the possibility of drug-induced pancreatitis.

This is the first report of *C. psittaci* pneumonia complicated by acute pancreatitis, diagnosed via mNGS. We demonstrated that mNGS can be used for the timely diagnosis of *C. psittaci* pneumonia. *C. psittaci* infection may also lead to acute pancreatitis, however, further case reports are required to support this conclusion. In addition, early, targeted, anti-infective and supportive therapies are essential to achieve favorable clinical outcomes.

## Data availability statement

The datasets presented in this article are not readily available because of ethical/privacy restrictions. Requests to access the datasets should be directed to the corresponding author.

## Ethics statement

The studies involving humans were approved by Huizhou Central People’s Hospital. The studies were conducted in accordance with the local legislation and institutional requirements. The participants provided their written informed consent to participate in this study. Written informed consent was obtained from the individual(s) for the publication of any potentially identifiable images or data included in this article.

## Author contributions

CF: Writing – original draft, Funding acquisition. YX: Data curation, Writing – original draft. HM: Formal analysis, Methodology, Writing – original draft. LX: Writing – original draft.
